# Supplementary material for: Transcriptional Regulatory Network of GA Floral Induction Pathway in LA Hybrid Lily
Source: Int J Mol Sci. 2019 May 31;20(11):2694. doi: 10.3390/ijms20112694 (PMC6600569; doi:10.3390/ijms20112694)
Supplement: Supplementary file 1 [file ijms-20-02694-s001.pdf]

Table 1S. The KEGG biochemical pathways categorization of LA lily unigenes pathways.

KEGG Categories	Mapped-KO	Unigene-NUM	Ratio of No.	ALL pathway KO	Pathway-ID
Metabolic pathways	954	4337	8.66	2067	ko01100
Biosynthesis of secondary metabolites	403	2285	4.56	720	ko01110
Biosynthesis of antibiotics	206	1147	2.29	---	ko01130
Microbial metabolism in diverse environments	157	995	1.99	720	ko01120
Ribosome	122	504	1.01	142	ko03010
Spliceosome	107	502	1	115	ko03040
Biosynthesis of amino acids	105	614	1.23	---	ko01230
Carbon metabolism	104	707	1.41	---	ko01200
Oxidative phosphorylation	100	335	0.67	206	ko00190
Purine metabolism	100	386	0.77	237	ko00230
RNA transport	98	861	1.72	134	ko03013
Endocytosis	92	736	1.47	138	ko04144
Protein processing in endoplasmic reticulum	88	567	1.13	137	ko04141
homologous recombination	84	933	1.86	144	ko05169
Ubiquitin mediated proteolysis	78	396	0.79	119	ko04120
HTLV-I infection	76	367	0.73	199	ko05166
Pyrimidine metabolism	76	298	0.6	150	ko00240
Non-alcoholic fatty liver disease (NAFLD)	72	209	0.42	---	ko04932
PI3K-Akt signaling pathway	71	328	0.66	226	ko04151
Viral carcinogenesis	68	387	0.77	132	ko05203
Cell cycle	63	339	0.68	103	ko04110
Proteoglycans in cancer	58	229	0.46	---	ko05205
Regulation of actin cytoskeleton	58	234	0.47	144	ko04810
Ribosome biogenesis in eukaryotes	56	237	0.47	82	ko03008
Phagosome	54	280	0.56	93	ko04145
Focal adhesion	53	185	0.37	133	ko04510
Lysosome	53	253	0.51	99	ko04142
RNA degradation	53	305	0.61	70	ko03018
Herpes simplex infection	52	257	0.51	121	ko05168
Cell cycle - yeast	51	260	0.52	118	ko04111
MAPK signaling pathway	50	164	0.33	181	ko04010
mRNA surveillance pathway	49	338	0.68	59	ko03015
mTOR signaling pathway	47	177	0.35	30	ko04150
Peroxisome	47	229	0.46	70	ko04146
MicroRNAs in cancer	45	224	0.45	---	ko05206
AMPK signaling pathway	45	295	0.59	---	ko04152
Insulin signaling pathway	44	259	0.52	77	ko04910
Glycine, serine and threonine metabolism	44	449	0.9	69	ko00260
Cysteine and methionine metabolism	44	245	0.49	64	ko00270
Oxytocin signaling pathway	42	256	0.51	---	ko04921
Influenza A	41	244	0.49	109	ko05164
Starch and sucrose metabolism	41	465	0.93	79	ko00500
Photosynthesis	41	119	0.24	62	ko00195
Fatty acid metabolism	41	191	0.38	---	ko01212
Amino sugar and nucleotide sugar metabolism	40	280	0.56	107	ko00520
Rap1 signaling pathway	40	134	0.27	---	ko04015
FoxO signaling pathway	40	209	0.42	---	ko04068
Oocyte meiosis	39	268	0.54	74	ko04114
Meiosis - yeast	39	220	0.44	99	ko04113
Others	4639	25,820	51.6		

Table 2S. The KEGG biochemical pathways categorization of 2,148 DEGs pathways in *LA* lily.

	Pathway	DEGs genes with pathway annotation (183)		All genes with pathway annotation (6343)	Pvalue	Qvalue	Pathway ID
**1**	Amino sugar and nucleotide sugar metabolism	16	8.74%	277 (4.37%)	0.006053	0.055818	ko00520
2	Glycolysis / Gluconeogenesis	16	8.74%	361 (5.69%)	0.056356	0.259865	ko00010
3	Phenylpropanoid biosynthesis	13	7.10%	221 (3.48%)	0.011194	0.09291	ko00940
4	Starch and sucrose metabolism	13	7.10%	452 (7.13%)	0.547199	0.99969	ko00500
5	Biosynthesis of amino acids	13	7.10%	622 (9.81%)	0.920497	0.99969	ko01230
6	Carbon metabolism	13	7.10%	701 (11.05%)	0.973615	0.99969	ko01200
7	alpha-Linolenic acid metabolism	12	6.56%	88 (1.39%)	0.000007	0.000291	ko00592
8	Flavonoid biosynthesis	11	6.01%	55 (0.87%)	0	0.000028	ko00941
9	Glutathione metabolism	11	6.01%	187 (2.95%)	0.018977	0.131256	ko00480
10	Phenylalanine metabolism	10	5.46%	93 (1.47%)	0.000317	0.004321	ko00360
11	Glycerophospholipid metabolism	10	5.46%	201 (3.17%)	0.064739	0.282806	ko00564
12	Plant-pathogen interaction	9	4.92%	155 (2.44%)	0.034747	0.205998	ko04626
13	Circadian rhythm - plant	8	4.37%	42 (0.66%)	0.000021	0.00058	ko04712
14	Zeatin biosynthesis	8	4.37%	46 (0.73%)	0.000042	0.000698	ko00908
15	Fatty acid degradation	8	4.37%	154 (2.43%)	0.076485	0.3023	ko00071
16	Flavone and flavonol biosynthesis	7	3.83%	33 (0.52%)	0.000034	0.000696	ko00944
17	Ubiquinone and other terpenoid-quinone biosynthesis	7	3.83%	65 (1.02%)	0.002514	0.026078	ko00130
18	Plant hormone signal transduction	7	3.83%	114 (1.8%)	0.046075	0.254947	ko04075
19	Pentose and glucuronate interconversions	7	3.83%	118 (1.86%)	0.053723	0.259865	ko00040
20	Cysteine and methionine metabolism	7	3.83%	249 (3.93%)	0.583228	0.99969	ko00270
21	Degradation of aromatic compounds	6	3.28%	34 (0.54%)	0.000364	0.004321	ko01220
22	Tyrosine metabolism	6	3.28%	101 (1.59%)	0.070999	0.294647	ko00350
23	Tryptophan metabolism	5	2.73%	106 (1.67%)	0.190797	0.609082	ko00380
24	Galactose metabolism	5	2.73%	179 (2.82%)	0.593533	0.99969	ko00052
25	Carbon fixation in photosynthetic organisms	5	2.73%	204 (3.22%)	0.70743	0.99969	ko00710
26	Purine metabolism	5	2.73%	340 (5.36%)	0.971853	0.99969	ko00230
27	Linoleic acid metabolism	4	2.19%	49 (0.77%)	0.051915	0.259865	ko00591
28	Diterpenoid biosynthesis	4	2.19%	66 (1.04%)	0.12222	0.422676	ko00904
29	Phenylalanine, tyrosine and tryptophan biosynthesis	4	2.19%	102 (1.61%)	0.33958	0.939504	ko00400
30	Glycerolipid metabolism	4	2.19%	157 (2.48%)	0.66959	0.99969	ko00561
31	Fatty acid metabolism	4	2.19%	195 (3.07%)	0.82059	0.99969	ko01212
32	Pyruvate metabolism	4	2.19%	258 (4.07%)	0.944755	0.99969	ko00620
33	Stilbenoid, diarylheptanoid and gingerol biosynthesis	3	1.64%	19 (0.3%)	0.016287	0.12289	ko00945
34	C5-Branched dibasic acid metabolism	3	1.64%	24 (0.38%)	0.030631	0.195568	ko00660
35	Carotenoid biosynthesis	3	1.64%	52 (0.82%)	0.188786	0.609082	ko00906
36	Valine, leucine and isoleucine biosynthesis	3	1.64%	60 (0.95%)	0.249535	0.739694	ko00290
37	Biosynthesis of unsaturated fatty acids	3	1.64%	64 (1.01%)	0.280961	0.804128	ko01040
38	N-Glycan biosynthesis	3	1.64%	79 (1.25%)	0.399939	0.99969	ko00510
39	Pentose phosphate pathway	3	1.64%	130 (2.05%)	0.730232	0.99969	ko00030
40	Alanine, aspartate and glutamate metabolism	3	1.64%	132 (2.08%)	0.739726	0.99969	ko00250
41	Phosphatidylinositol signaling system	3	1.64%	151 (2.38%)	0.817213	0.99969	ko04070
42	Fructose and mannose metabolism	3	1.64%	155 (2.44%)	0.830792	0.99969	ko00051
43	2-Oxocarboxylic acid metabolism	3	1.64%	157 (2.48%)	0.837253	0.99969	ko01210
44	Insulin resistance	3	1.64%	158 (2.49%)	0.840404	0.99969	ko04931
45	Citrate cycle (TCA cycle)	3	1.64%	185 (2.92%)	0.907644	0.99969	ko00020
46	Aminoacyl-tRNA biosynthesis	3	1.64%	190 (3%)	0.916851	0.99969	ko00970
47	Pyrimidine metabolism	3	1.64%	252 (3.97%)	0.979075	0.99969	ko00240
48	Taurine and hypotaurine metabolism	2	1.09%	21 (0.33%)	0.121644	0.422676	ko00430
49	AGE-RAGE signaling pathway in diabetic complications	2	1.09%	32 (0.5%)	0.235481	0.723887	ko04933
50	Ether lipid metabolism	2	1.09%	61 (0.96%)	0.52958	0.99969	ko00565
51	mRNA surveillance pathway	2	1.09%	92 (1.45%)	0.749662	0.99969	ko03015
52	Endocytosis	2	1.09%	96 (1.51%)	0.770505	0.99969	ko04144
53	Ascorbate and aldarate metabolism	2	1.09%	99 (1.56%)	0.785136	0.99969	ko00053
54	Propanoate metabolism	2	1.09%	103 (1.62%)	0.803371	0.99969	ko00640
55	Terpenoid backbone biosynthesis	2	1.09%	107 (1.69%)	0.820227	0.99969	ko00900
56	Fatty acid biosynthesis	2	1.09%	108 (1.7%)	0.824234	0.99969	ko00061
57	Arginine and proline metabolism	2	1.09%	131 (2.07%)	0.896784	0.99969	ko00330
58	Valine, leucine and isoleucine degradation	2	1.09%	149 (2.35%)	0.933015	0.99969	ko00280

59	Glycine, serine and threonine metabolism	2	1.09%	170 (2.68%)	0.960121	0.99969	ko00260
60	Oxidative phosphorylation	2	1.09%	171 (2.7%)	0.961107	0.99969	ko00190
61	Peroxisome	2	1.09%	183 (2.89%)	0.971261	0.99969	ko04146
62	Vancomycin resistance	1	0.55%	3 (0.05%)	0.084092	0.317256	ko01502
63	Mismatch repair	1	0.55%	20 (0.32%)	0.44367	0.99969	ko03430
64	Vitamin B6 metabolism	1	0.55%	25 (0.39%)	0.519672	0.99969	ko00750
65	Thiamine metabolism	1	0.55%	26 (0.41%)	0.533584	0.99969	ko00730
66	Biotin metabolism	1	0.55%	32 (0.5%)	0.609033	0.99969	ko00780
67	Regulation of autophagy	1	0.55%	41 (0.65%)	0.700049	0.99969	ko04140
68	Lysine biosynthesis	1	0.55%	43 (0.68%)	0.717217	0.99969	ko00300
69	Nicotinate and nicotinamide metabolism	1	0.55%	46 (0.73%)	0.741155	0.99969	ko00760
70	Selenocompound metabolism	1	0.55%	47 (0.74%)	0.748677	0.99969	ko00450
71	Fatty acid elongation	1	0.55%	51 (0.8%)	0.776654	0.99969	ko00062
72	Nucleotide excision repair	1	0.55%	53 (0.84%)	0.789458	0.99969	ko03420
73	Histidine metabolism	1	0.55%	57 (0.9%)	0.812916	0.99969	ko00340
74	Other glycan degradation	1	0.55%	64 (1.01%)	0.847884	0.99969	ko00511
75	Sulfur metabolism	1	0.55%	70 (1.1%)	0.872629	0.99969	ko00920
76	Sphingolipid metabolism	1	0.55%	75 (1.18%)	0.89016	0.99969	ko00600
77	DNA replication	1	0.55%	80 (1.26%)	0.90529	0.99969	ko03030
78	Base excision repair	1	0.55%	87 (1.37%)	0.923051	0.99969	ko03410
79	Spliceosome	1	0.55%	112 (1.77%)	0.963421	0.99969	ko03040
80	Cyanoamino acid metabolism	1	0.55%	112 (1.77%)	0.963421	0.99969	ko00460
81	Inositol phosphate metabolism	1	0.55%	154 (2.43%)	0.989584	0.99969	ko00562
82	Protein processing in endoplasmic reticulum	1	0.55%	176 (2.77%)	0.994625	0.99969	ko04141
83	Ubiquitin mediated proteolysis	1	0.55%	270 (4.26%)	0.99969	0.99969	ko04120

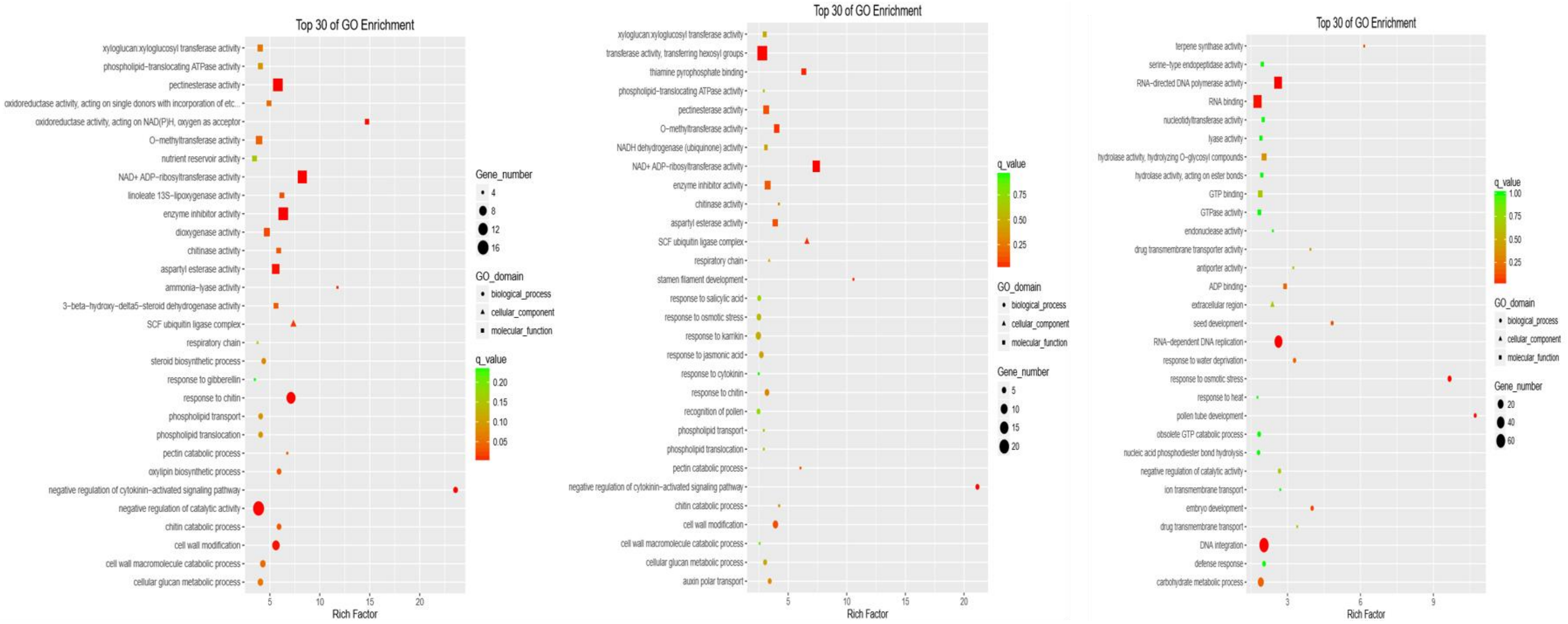

Figure 1S. Functional GO enrichment analysis of 2,148 DEGs

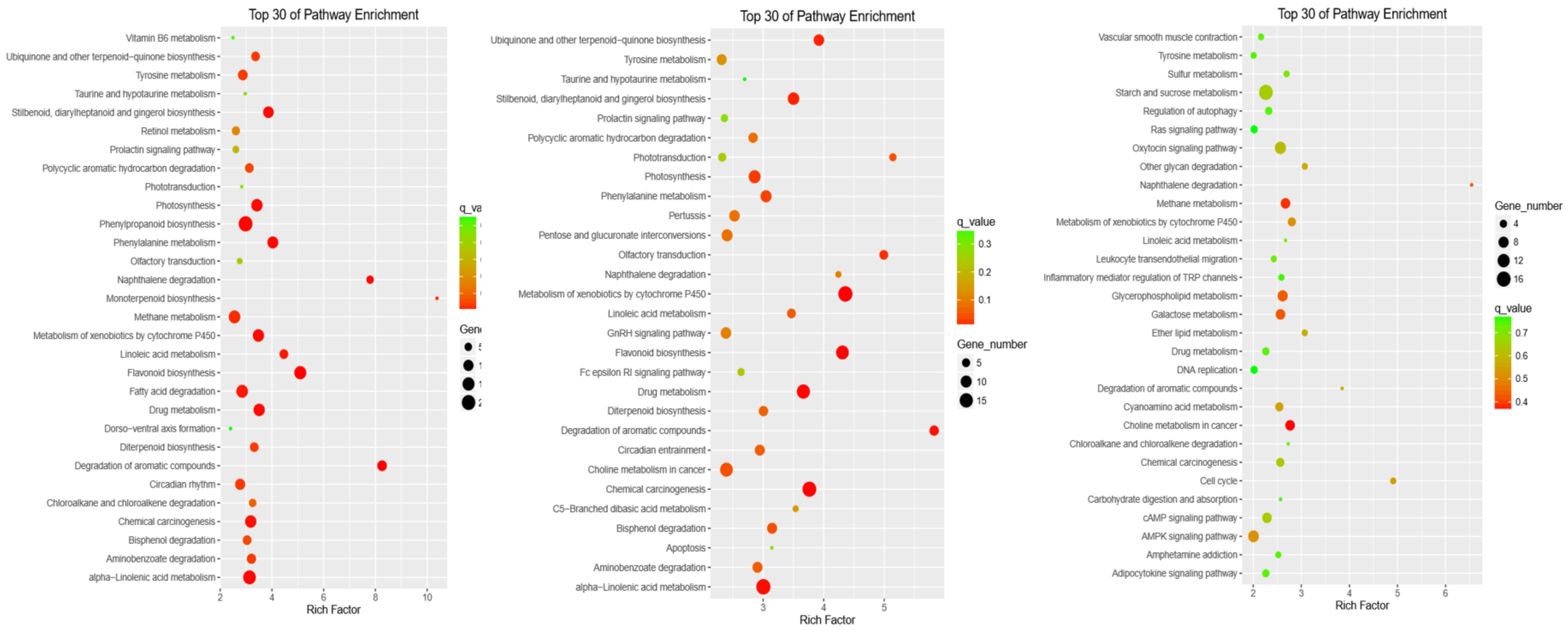

Figure 2S. Functional KEGG enrichment analysis of 2,148 DEGs.

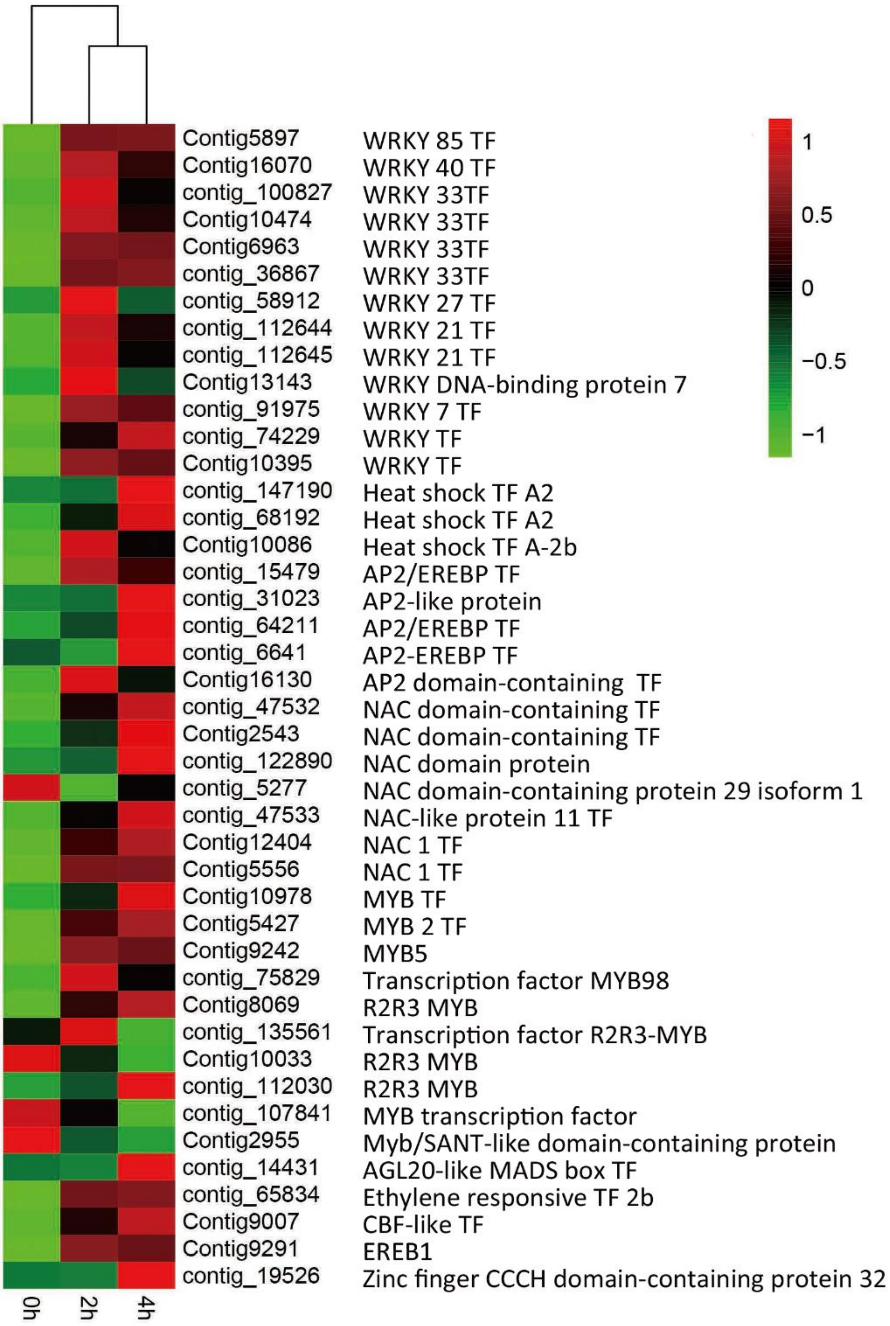

Figure 3S. Heat-map of TF.

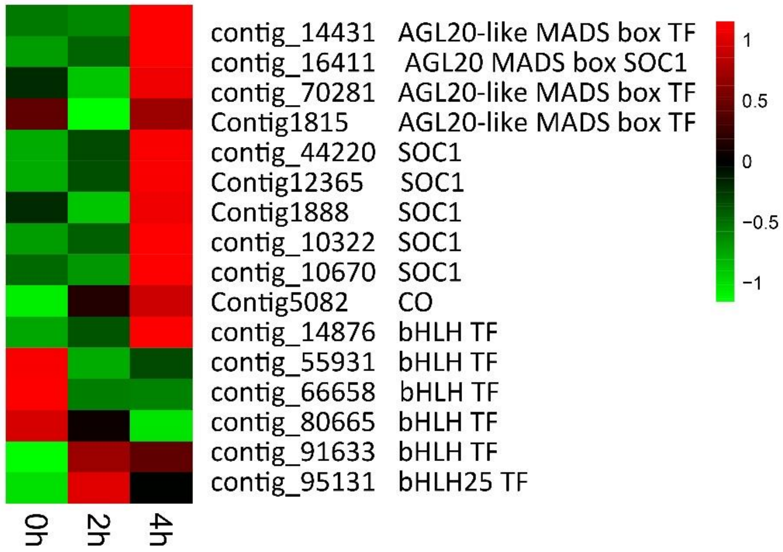

Figure 4S. Heat-map of floral transition genes.

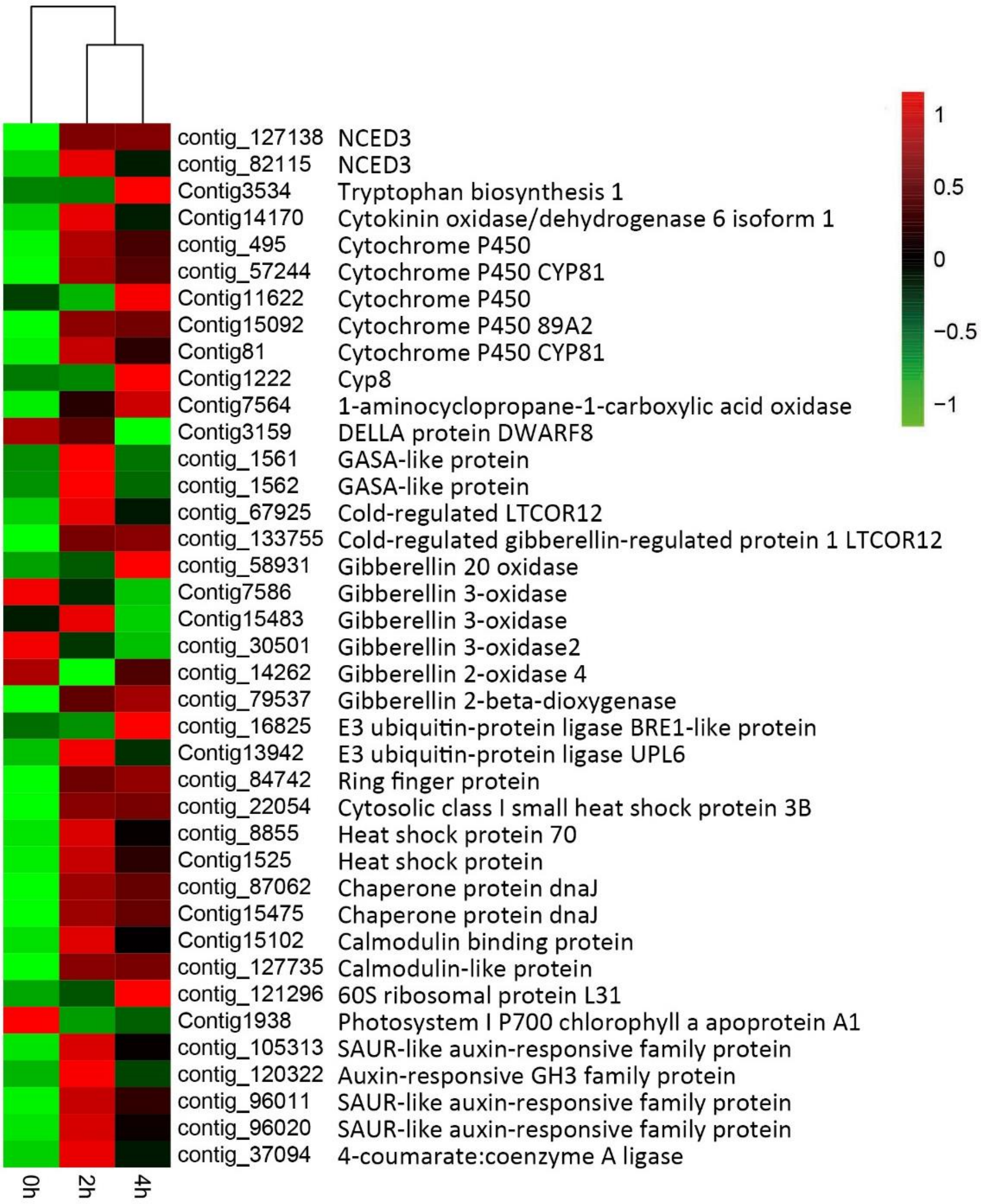

Figure 5S. Heat-map of hormone related genes.

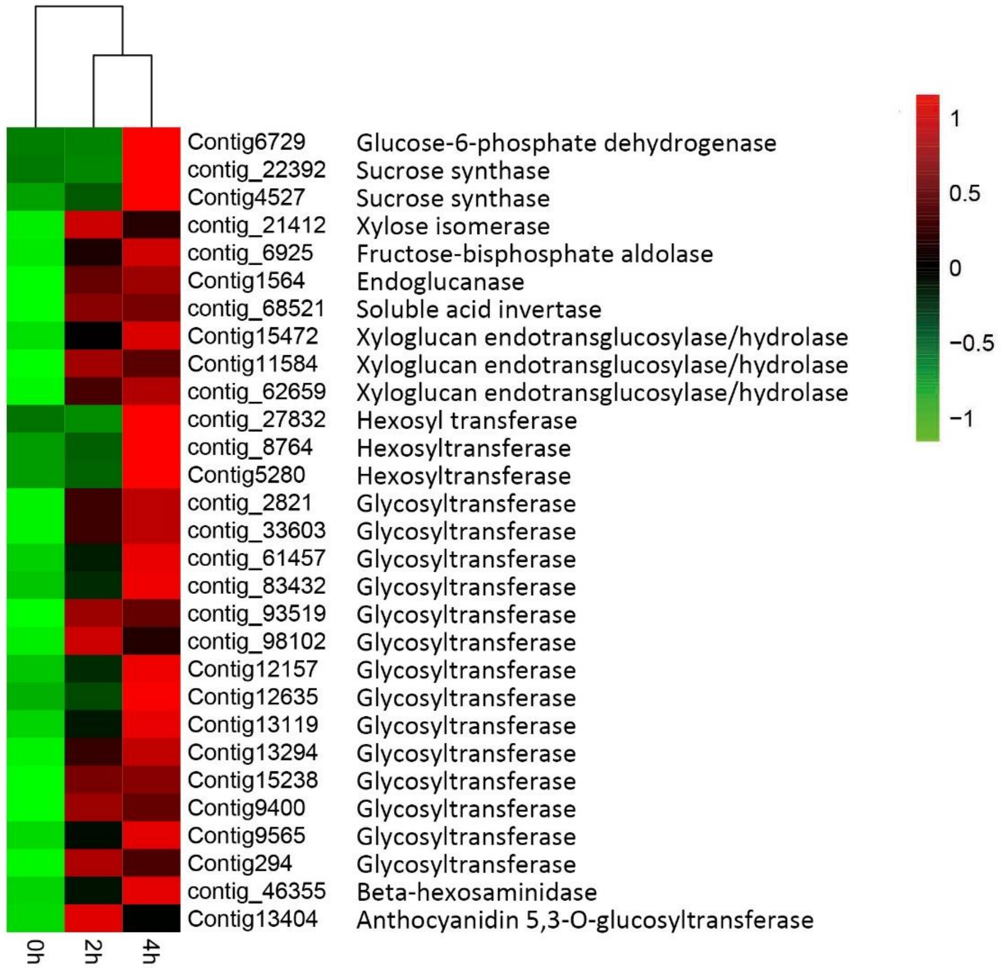

Figure 6S. Heat-map of carbohydrate related genes.

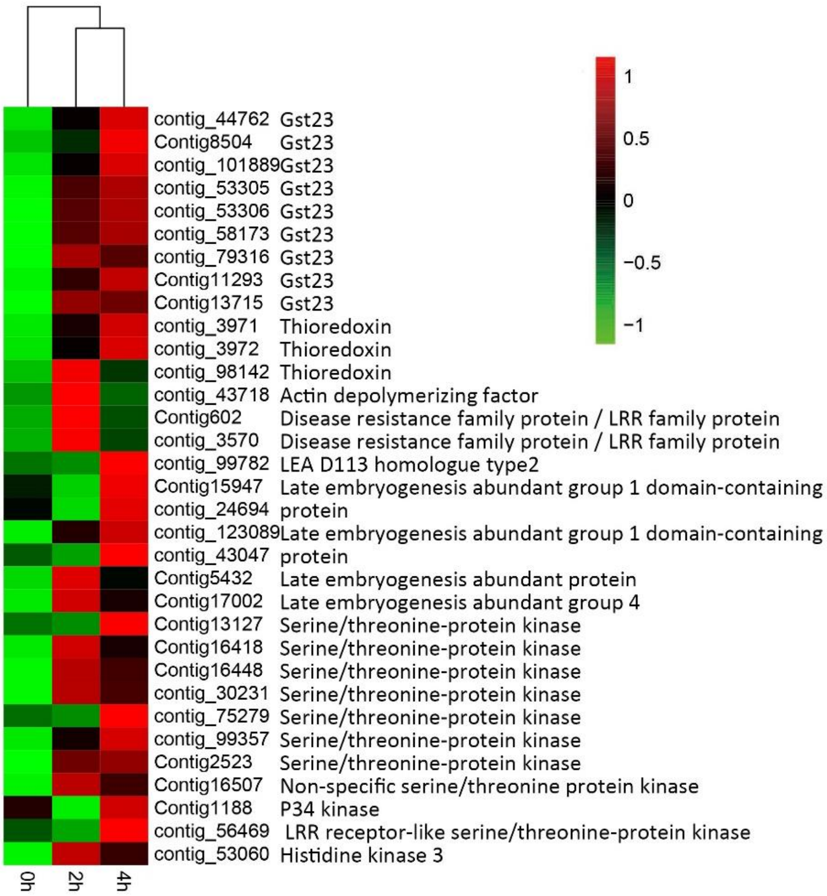

Figure 7S. Heat-map of resistance genes.

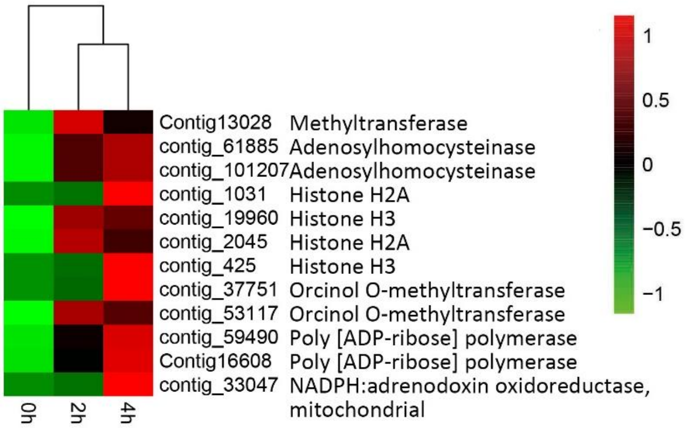

Figure 8S. Heat-map of apparent modification genes.

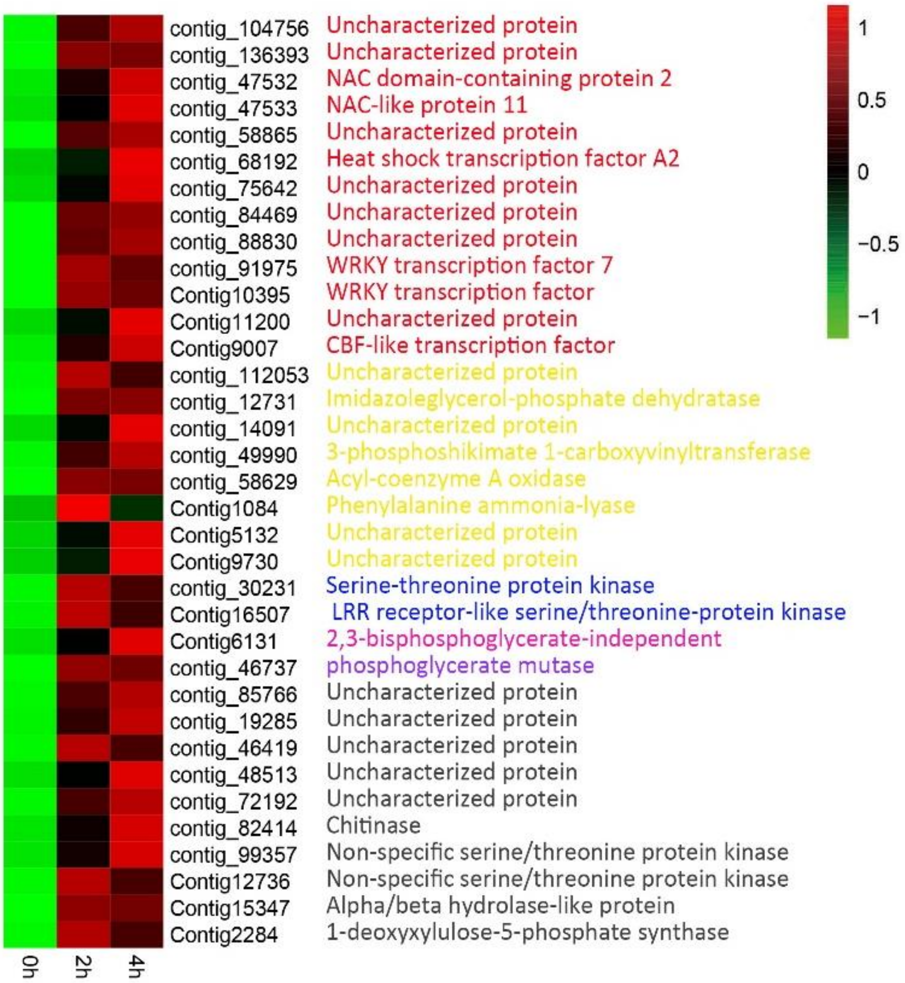

Figure 9S. Heatmap of genes in Module1

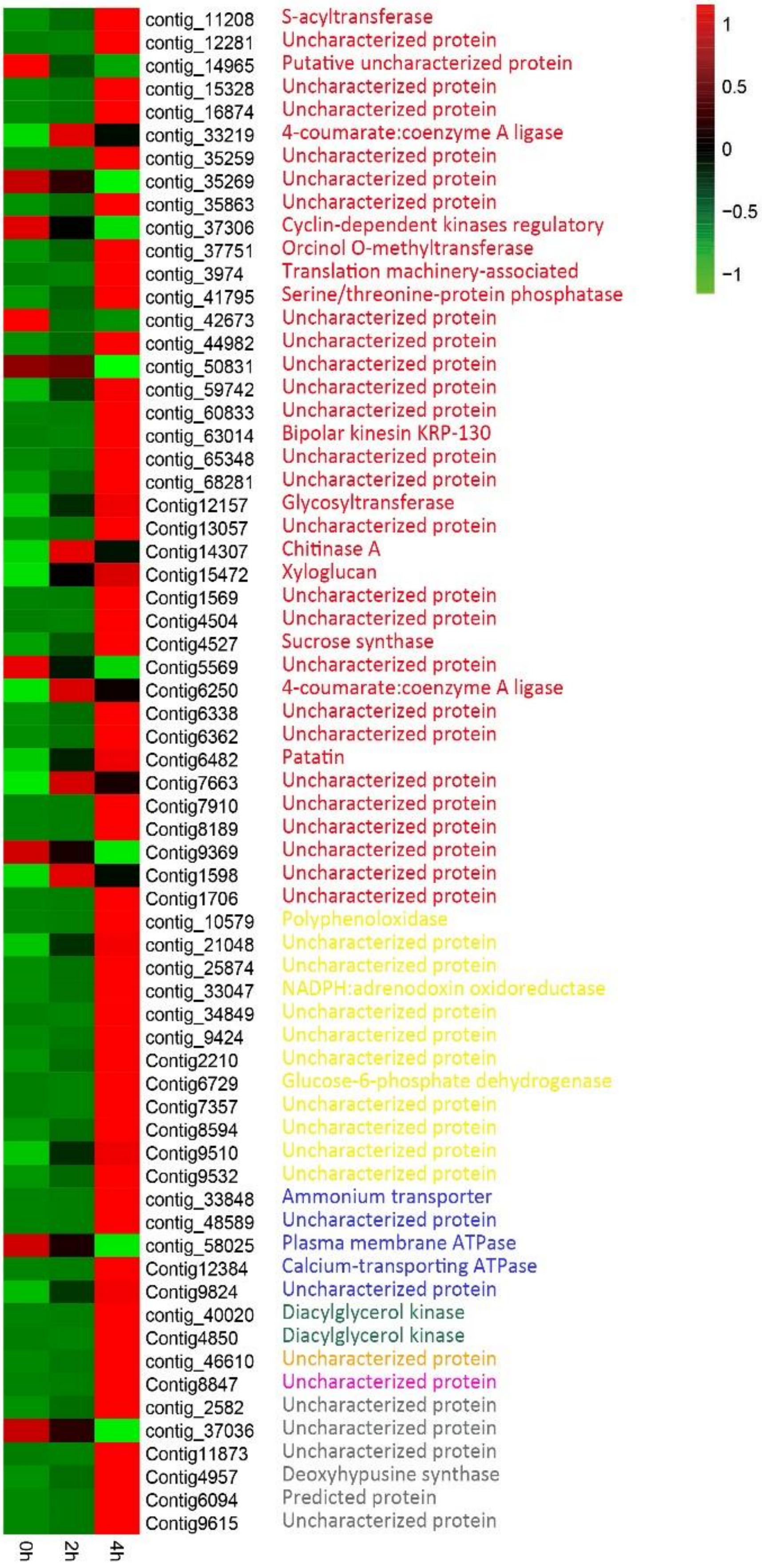

Figure 10S. Heatmap of genes in Module2.

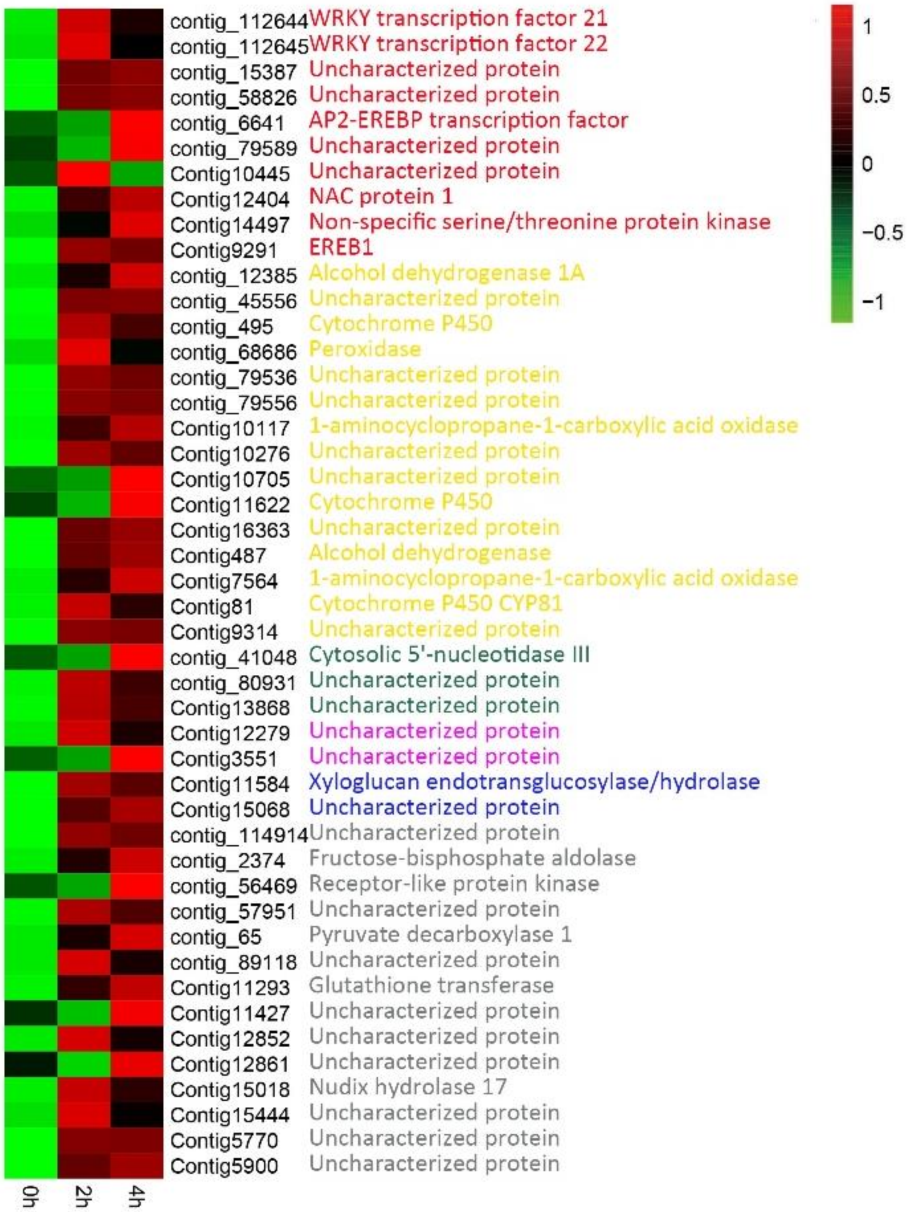

Figure 11S. Heatmap of genes in Module3.

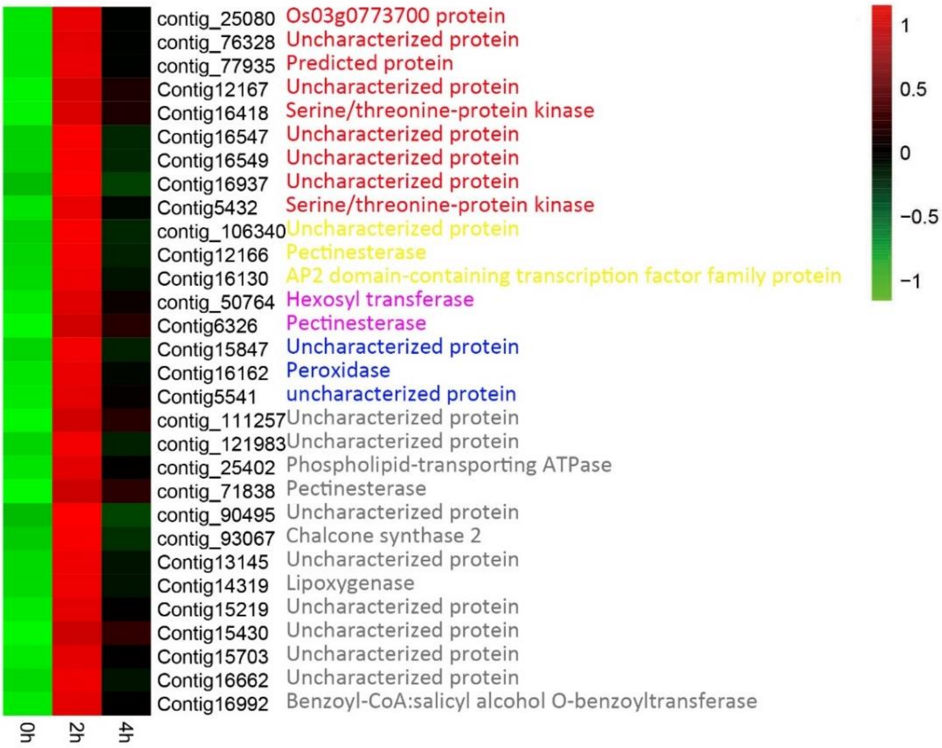

Figure 12S. Heatmap of genes in Module5.

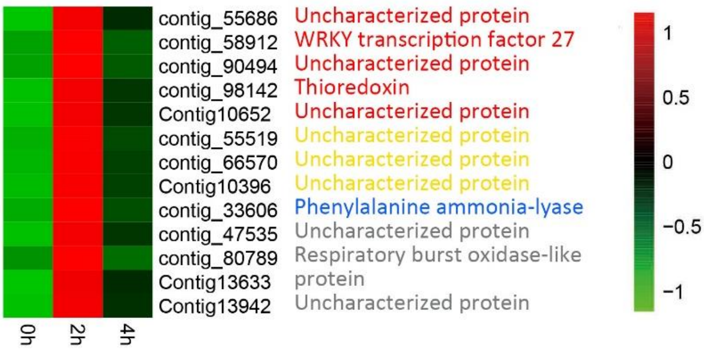

Figure 13S. Heatmap of genes in Module12.

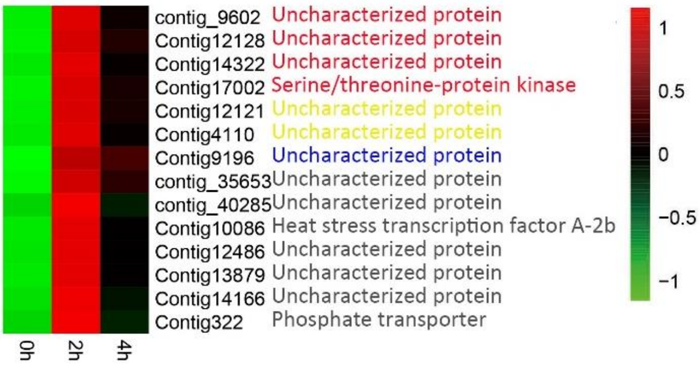

Figure 14S. Heatmap of genes in Module14.
